# Modeling Patient-Specific Muscular Dystrophy Phenotypes and Therapeutic Responses in Reprogrammed Myotubes Engineered on Micromolded Gelatin Hydrogels

**DOI:** 10.3389/fcell.2022.830415

**Published:** 2022-04-06

**Authors:** Florian Barthélémy, Jeffrey W. Santoso, Laura Rabichow, Rongcheng Jin, Isaiah Little, Stanley F. Nelson, Megan L. McCain, M. Carrie Miceli

**Affiliations:** ^1^ Department of Microbiology Immunology and Molecular Genetics, University of California, Los Angeles, Los Angeles, CA, United States; ^2^ Center for Duchenne Muscular Dystrophy, University of California, Los Angeles, Los Angeles, CA, United States; ^3^ Laboratory for Living Systems Engineering, Department of Biomedical Engineering, USC Viterbi School of Engineering, University of Southern California, Los Angeles, CA, United States; ^4^ Department of Neurology, David Geffen School of Medicine, University of California, Los Angeles, Los Angeles, CA, United States; ^5^ Department of Pathology and Laboratory Medicine, David Geffen School of Medicine, University of California, Los Angeles, Los Angeles, CA, United States; ^6^ Department of Stem Cell Biology and Regenerative Medicine, Keck School of Medicine of USC, University of Southern California, Los Angeles, CA, United States

**Keywords:** DMD, LGMD, exon skipping, hydrogels, calpain 3, dystrophin

## Abstract

*In vitro* models of patient-derived muscle allow for more efficient development of genetic medicines for the muscular dystrophies, which often present mutation-specific pathologies. One popular strategy to generate patient-specific myotubes involves reprogramming dermal fibroblasts to a muscle lineage through MyoD induction. However, creating physiologically relevant, reproducible tissues exhibiting multinucleated, aligned myotubes with organized striations is dependent on the introduction of physicochemical cues that mimic the native muscle microenvironment. Here, we engineered patient-specific control and dystrophic muscle tissues *in vitro* by culturing and differentiating MyoD–directly reprogrammed fibroblasts isolated from one healthy control subject, three patients with Duchenne muscular dystrophy (DMD), and two Limb Girdle 2A/R1 (LGMD2A/R1) patients on micromolded gelatin hydrogels. Engineered DMD and LGMD2A/R1 tissues demonstrated varying levels of defects in α-actinin expression and organization relative to control, depending on the mutation. In genetically relevant DMD tissues amenable to mRNA reframing by targeting exon 44 or 45 exclusion, exposure to exon skipping antisense oligonucleotides modestly increased myotube coverage and alignment and rescued dystrophin protein expression. These findings highlight the value of engineered culture substrates in guiding the organization of reprogrammed patient fibroblasts into aligned muscle tissues, thereby extending their value as tools for exploration and dissection of the cellular and molecular basis of genetic muscle defects, rescue, and repair.

## Introduction

Muscular dystrophies are a heterogenous group of genetic disorders characterized by progressive muscle weakness. Duchenne (DMD-OMIM310200) and Limb Girdle 2A/R1 (LGMD2A/R1-OMIM 253600) muscular dystrophies are caused by mutations in the *DMD* and *CAPN3* genes, respectively. *DMD* generally presents early in childhood and results in loss of ambulation in early teenage years and fatal cardiac or respiratory insufficiencies in their twenties (Flanigan, 2014). LMGD2A/R1 has a more variable presentation, with onset of muscle weakness ranging between 0 and 40 years of age and different rates of progression (Kramerova et al., 2007). *DMD*-encoded dystrophin and *CAPN3*-encoded calpain proteins have both been implicated in contributing to the structural integrity and maintenance of the sarcomere and muscle health (Blau et al., 1983; Muntoni, 2001; Kramerova et al., 2007; van der Wal et al., 2018; Pakula et al., 2019; Verhaart et al., 2019; Caputo et al., 2020; Ortiz-Cordero et al., 2021). Therefore, culture platforms that enable sarcomere maturation may help in elucidating the molecular basis of dystrophin and calpain 3 contributions to the organization and maturation of striated skeletal muscle. Whereas molecular characterization of these dystrophies has led to the development of mutation specific genetic medicines, few human mutationally defined pre-clinical cell models suitable for testing of drugs with target mutation subsets have been developed.

A major challenge in developing culture models for muscular dystrophies and other rare muscle diseases is cell source. Primary myoblasts require access to fresh muscle tissue, have limited capacity to divide, and often require purification (Blau et al., 1983; Pakula et al., 2019). Because most diagnoses are now made through genetic testing, biopsies of muscular dystrophy patients are rarely performed, and fresh muscle biopsy tissue is largely unavailable (Muntoni, 2001; Verhaart et al., 2019). Alternatively, dermal fibroblasts derived from patient skin punches can be reprogrammed to induced pluripotent stem cells (iPSCs) and subsequently differentiated to myoblasts (van der Wal et al., 2018). Although iPSC technology has led to useful models for DMD and other muscle disorders (Caputo et al., 2020; Ortiz-Cordero et al., 2021), iPSCs can be difficult and costly to derive and expand (Speciale et al., 2020). A simpler approach is to reprogram dermal fibroblasts directly to myoblasts by expression of MyoD, a master regulator of muscle cell development. We and others have stably expressed an inducible MyoD construct in dermal fibroblasts partially immortalized through expression of human telomerase reverse transcriptase (hTERT), termed iDRMs (induced directly reprogrammed myotubes), which can be expanded as fibroblasts and then reprogrammed to myoblasts and multi-nucleated myotube-like cells upon MyoD induction (Chaouch et al., 2009; Kendall et al., 2012; Kim et al., 2016; Wein et al., 2017; Barthélémy et al., 2019). We previously used iDRM from patients with DMD on standard culture dishes to evaluate exon skipping strategies aimed at dystrophin rescue by reframing the RNA through exclusion of an additional exon (Wein et al., 2014; Barthélémy et al., 2018; Barthélémy et al., 2019; Gibbs et al., 2019). Although we were able to detect low levels of rescued dystrophin protein for certain DMD patient lines (Barthélémy et al., 2019), iDRMs were randomly organized and rarely survived longer than 1 week due to cell death and delamination, which may not be sufficient to detect dystrophin rescue in all patient lines.

To overcome some limitations of conventional *in vitro* techniques, several approaches to engineer DMD muscle tissue have been developed (Santoso and McCain, 2020). Fibronectin has been microcontact-printed as line patterns onto elastomeric polydimethylsiloxane (PDMS) substrates that were then laser-engraved into cantilevers to measure force generation. Primary control and DMD myoblasts seeded on these surfaces fused into aligned myotubes, with DMD myotubes exhibiting less actin and nuclear alignment and therefore lower contractile stress (Nesmith et al., 2016). However, PDMS is far stiffer than native muscle tissues, which is thought to contribute to delamination of cell line-derived myotubes after approximately 1 week in culture (Bettadapur et al., 2016a). Three-dimensional (3-D) muscle tissues have also been engineered by mixing myoblasts in matrix-derived hydrogels and allowing the tissue to compact and align across two anchor points, providing a closer match to the compliance of native tissue and a more relevant microenvironment. 3-D tissues engineered with DMD iPSC-derived myoblasts have demonstrated nuclear, cytoskeletal, and contractile abnormalities (Maffioletti et al., 2018; Ebrahimi et al., 2021). However, 3-D tissues models require high numbers of cells and generally entail complex fabrication procedures, limiting their overall throughput.

Micromolded gelatin hydrogels have been developed by us and others as substrates for lengthening the culture lifetime and improving the alignment and maturation of myotubes differentiated from C2C12 (Bettadapur et al., 2016a; Denes et al., 2019a), primary chick (Santoso et al., 2021a; Gupta et al., 2021), and human control and DMD iPSC-derived myoblasts (Al Tanoury et al., 2021). These gelatin substrates are relatively inexpensive and easy to fabricate while also matching the compliance of native muscle tissue. Here, we cultured iDRM control and DMD patient-derived myotubes on micromolded gelatin hydrogels to promote their alignment and stability. We demonstrate that iDRM on the hydrogels align and form myofiber-like structures with different degrees of maturity, evaluated with α-actinin immunostaining and confocal microscopy. Moreover, we treated tissues with Antisense oligonucleotid-directed exon skipping molecules and evaluated dystrophin gene and protein expression. We also engineered tissues from two newly derived LGMD2A/R1 iDRM, demonstrating the application of these approaches to other rare muscle diseases. Together, these findings highlight the value of combining patient-derived reprogrammed cell models with engineered substrates to evaluate and screen personalized therapies for rare muscle diseases.

## Material and Methods

### iDRM Derivation and Dermal Fibroblast Donor Characteristics

MyoD-reprogrammed skin punch fibroblasts (iDRM) were derived from three DMD subjects (Wang et al., 2018; Gibbs et al., 2019). As reported, iDRM derived from DMD subject 1003, who lost ambulation at 13.5 years, has an out-of-frame deletion of *DMD* exons 46–51 and is predicted not to express the dystrophin protein (Wang et al., 2018). DMD subject 1023 has an in-frame deletion of *DMD* exons 3 to 23 with sub-typical, albeit significant, sarcolemmal dystrophin protein expression (Gibbs et al., 2019) and remains ambulatory at age 20. Subject 1015 lost ambulation at age 15 and harbors a deletion of *DMD* exon 45, low levels of self-corrected exon 44 “skipped” mRNA in derived iDRM (Wang et al., 2018), and an increased frequency of clusters of dystrophin-positive revertant fibers. Muscle biopsies were only available from subjects 1015 and 1023 and a healthy 20-year-old female volunteer who served as a positive control for normal dystrophin expression and localization.

MyoD-reprogrammed skin fibroblasts (iDRM) from two LGMD2A/R1 were established for this study. Subject 1077 is a 52-year-old male who lost ambulation at 44 years old with compound heterozygous mutations: c.550delA (p.T184Rfs*36) and c.1342C > G (p448R > G) in *CAPN3*. Both mutations are predicted to be pathogenic (https://databases.lovd.nl/shared/genes/CAPN3). Subject 1081 is a 37-year-old female presenting a homozygous deletion of *CAPN3* exons 17 to 24 [ c.(1914 + 1_1915–1)_(*544_?) del]. Deletion of these exons leads to a short truncated non-functional *CAPN3* protein (BhattBHATT1 et al., 2019). All these variants leading to LGMD2A/R1 have been previously described in other patients (BhattBHATT1 et al., 2019; Krahn et al., 2006; Nallamilli et al., 2018).

Skin punches and muscle biopsies were obtained with informed consent from patients of the Center for Duchenne Muscular Dystrophy (CDMD) at UCLA under University of California Los Angeles Institutional Review Board (IRB)–approved protocols #11–001087 and #18–001547. Open or needle skeletal muscle biopsies were performed and processed as previously described (Barthelemy et al., 2020; Lee et al., 2020). Muscle samples were sectioned at 10 µm thickness and stained with hematoxylin and eosin for global histology assessment or dystrophin/laminin using standard immunohistochemistry (Barthelemy et al., 2020), where dystrophin NCL-2 (Leica, Buffalo Grove, IL, United States) at a dilution of 1:50 and laminin L9393 (Sigma, St. Louis, MO, United States) at a dilution of 1:25 were used. Digital histological images were acquired using standard light and fluorescence using an Axioplan 2 microscope (Carl Zeiss Inc., United States). Pictures were then processed with AxioVision software (Zeiss) and/or ImageJ software.

### Inducible Directly Reprogrammable Myotube (iDRM) Lines

**FIGURE 1:**
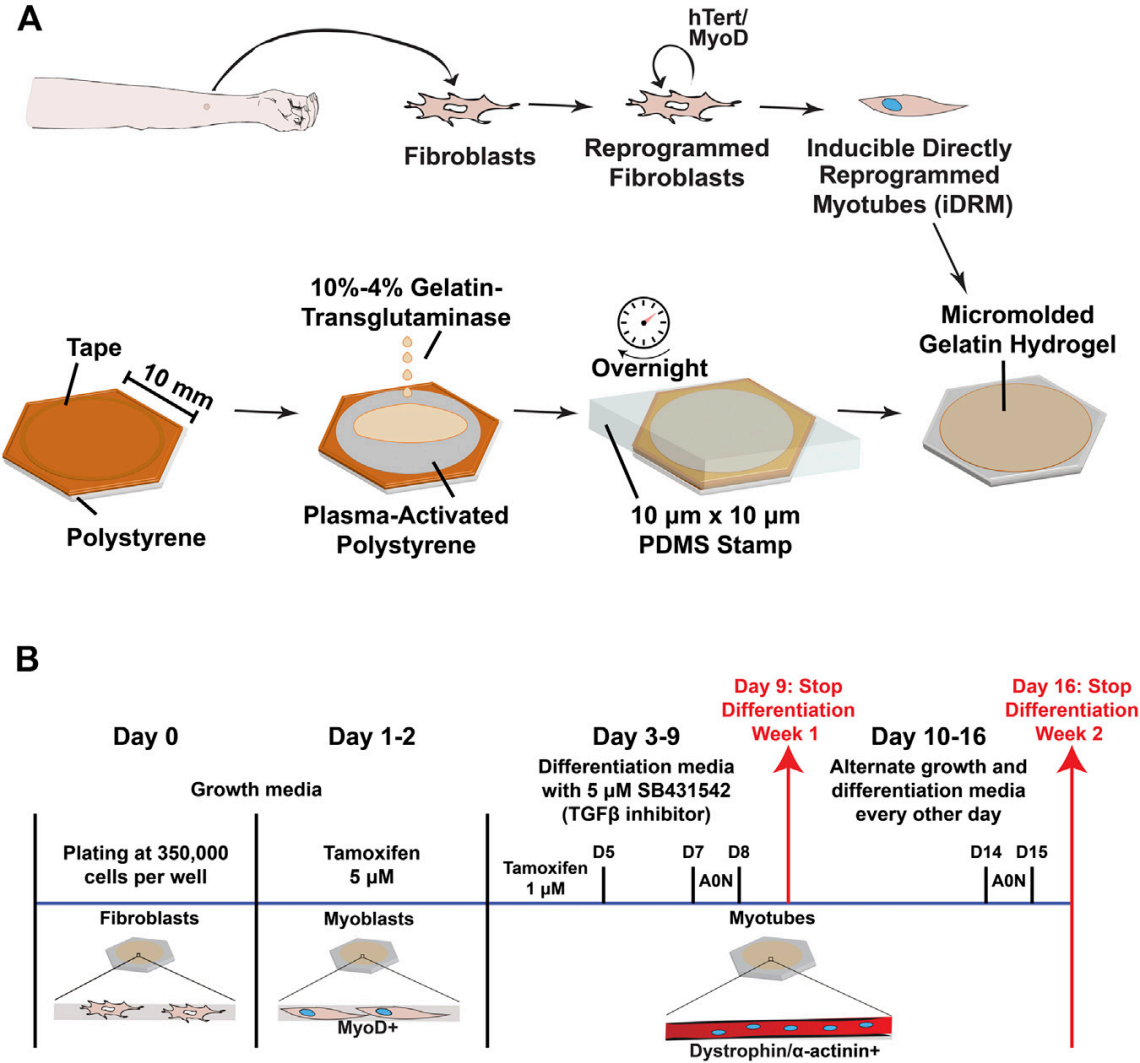
Overview of the experimental design and culture timeline. **(A)** A skin punch of 3 mm diameter is performed on the forearm and the tissue dissociated to obtain fibroblasts. Following infection with lentiviruses encoding hTERT and MyoD, cells (renamed induced directly reprogramed myotubes–iDRM) can form myotubes upon induction of MyoD- and fusion-inducing media. Polystyrene coverslips are plasma-treated to enhance gelatin hydrogel adhesion before molding of the surface using soft lithography adhesion before molding of the surface using soft lithography. Hydrogels are rehydrated and stored in PBS before cell seeding. **(B)** A typical timeframe for the generation of myotubes from iDRM with or without AO treatment. Analysis for experiments was performed after 1 (day 9) and 2 (day 16) weeks of differentiation.

iDRMs were generated from dermal fibroblasts cultured from skin punches of 3 mm diameter obtained from each participant. After the establishment of a fibroblast culture, cells were immortalized using a lentivirus encoding hTERT and subsequently infected with a lentivirus encoding a tamoxifen-inducible MyoD to allow commitment to skeletal muscle lineage, as we have previously reported for derivation of DMD 1023 and DMD 1015 iDRM (Barthélémy et al., 2019; Gibbs et al., 2019) (Figure 1A).

iDRMs were cultured as previously described, modified by plating on coverslips with micromolded gelatin hydrogels (described below) at the time of fibroblast plating, before MyoD induction (Figure 1B) (Barthelemy et al., 2018). Briefly, cells were kept in fibroblast growth media [DMEM (+ phenol red, high glucose) (Thermo Fisher Scientific, Grand Island, NY, United States) + 15% fetal bovine serum (Omega Scientific, Tarzana, CA, United States) + 1% nonessential amino acids (Thermo Fisher Scientific, Grand Island, NY, United States) + 1% penicillin/streptomycin (Thermo Fisher Scientific, Grand Island, NY, United States)]. To induce differentiation to myotubes, cells were first incubated in fibroblast growth media containing 5 µM of 4-OH tamoxifen (Sigma, St. Louis, MO, United States; dissolved in ethanol) for 48 h. On day 3, cells were washed in PBS (Thermo Fisher Scientific), and fusion media containing 1 μM 4-OH-tamoxifen was added [1:1 Ham’s F-10:DMEM (phenol red free, high glucose), 2% horse serum (Thermo Fisher Scientific, Grand Island, NY, United States), and 2% insulin-transferrin-selenium (Thermo Fisher Scientific, Grand Island, NY, United States)]. During the first week of differentiation, SB431542 (a TGF‐β inhibitor) was added at a final concentration of 5 µM. All lines were kept for up to 2 weeks in fusion conditions before analysis, and the medium was changed every other day (Figure 1B).

### Gelatin Hydrogel

The fabrication of micromolded gelatin hydrogels cross-linked with transglutaminase was performed as previously described (Santoso et al., 2021a; Gupta et al., 2021) and illustrated in Figure 1A. Briefly, 260-mm^2^ hexagons were cut from 150-mm polystyrene dishes and masked with tape. Inside each hexagonal coverslip, circles were laser-cut into the tape with a 30W Epilog Mini 24 Laser Engraver. Regions of the tape were removed such that only the outer border of substrates was covered. Coverslips were then treated with plasma (Harrick Plasma, Ithaca, NY, United States) for 10 min in ambient conditions to increase adherence of polystyrene.

PDMS stamps with 10-µm-wide grooves separated by 10 µm and 2 µm in height were manufactured using photolithography and soft lithography (Suh et al., 2017). Then, 20% porcine gelatin solution (Sigma, St. Louis, MO, United States) and 8% transglutaminase (Ajinomoto, Ontario, CA, United States) solutions in ultrapure water were mixed 1:1 with a centrifugal mixer (Thinky United States, Laguna Hills, CA, United States). The mixed solution (200 µl) was added to each coverslip, and stamps were slowly applied. Hydrogels were incubated at room temperature overnight to solidify. The next day, hydrogels were rehydrated. Stamps and remaining tape were carefully removed (Figure 1A). Substrates were washed and stored in PBS at 4°C until cell seeding.

### AO Transfection

On day 7 (week 1) or 14 (week 2) of differentiation, iDRM cells (1015 or 1003) were transfected with 25 or 250 nM 2-O-methyl AO targeting exon 44 (using H44A-TGTTCAGCTTCTGTTAGCCACTGA and 45 (using H45A -CCA​ATG​CCA​TCC​TGG​AGT​TCC​TGT​AA) (Wilton et al., 2007), respectively, using oligofectamine (Thermo Fisher Scientific) transfection reagent according to our previously published protocol (Barthélémy et al., 2019).

### RNA Isolation and PCR

On the day of analysis, duplicate or triplicate coverslips from each condition were removed from the well, and the remnant cells growing on the side of the coverslips were scraped directly and combined in TRIzol (Thermo Fisher Scientific). Total RNA was isolated using the Purelink RNA mini kit (Thermo Fisher Scientific). Exon skipping analysis was performed accordingly to our previously published protocol, and skipping efficiency is indicated by the ratio of skipped mRNA transcript over the total mRNA transcripts (skipped plus unskipped) (Barthélémy et al., 2019). For both cell lines, a nested PCR was performed to amplify the targeted DMD region: between exons 43 and 52 using the previously described primers for cell line CDMD1003 (Ex42-o, 5′-GTC​CGT​GAA​GAA​ACG​ATG​ATG-3′ + Ex53-0, 5′-CTC​CGG​TTC​TGA​AGG​TGT​TC-3′ and Ex43-i, 5′- TCT​CTC​CCA​GCT​TGA​TTT​CC-3′ and Ex52-i, 5′- TCT​AGC​CTC​TTG​ATT​GCT​GG-3′) or between exons 42 and 46 (Ex42-o, 5′-CAA​TGC​TCC​TGA​CCT​CTG​TGC-3′ + Ex46-o, 5′-GCT​CTT​TTC​CAG​GTT​CAA​GTG​G-3′ and Ex43-i, 5′-GTC​TAC​AAC​AAA​GCT​CAG​GTC​G-3′ + Ex46-i, 5′-GCA​ATG​TTA​TCT​GCT​TCC​TCC​AAC​C-3′) for cell line CDMD1015.

### Immunofluorescence Staining and Microscopy

iDRMs were seeded at 350,000 cells onto micromolded gelatin hydrogel coverslips in 12-well plates and MyoD was induced with tamoxifen as described above. During all procedures, cells were kept at room temperature unless specified otherwise. After myotube formation, cells were fixed with acetone for dystrophin staining or ice-cold methanol for α-actinin staining. Primary antibodies were incubated overnight at 4°C: dystrophin NCL-2 (Leica, Buffalo Grove, IL, United States) at a dilution of 1:20 and α-actinin (Sigma, St. Louis, MO, United States) at a dilution of 1:200. Secondary antibodies were used at a dilution of 1:500: goat anti-mouse IgG (SA5-10173) and goat anti-rabbit (35553) from Thermo Fisher Scientific for 1 h. Coverslips were mounted in ProLong Gold Antifade with DAPI (Thermo Fisher Scientific). Confocal fluorescence microscopy was performed using a Confocal Module Nikon C2 with 20× air or 60× oil objectives. Z-stacks were acquired (step size: 1 μm), and average intensity projections were used for data analysis on ImageJ (NIH).

To quantify myogenic index, CellProfiler was first used to mask α-actinin signal. Then, the proportion of total nuclei in masked areas that contained at least three nuclei was taken as the myogenic index; we chose to count areas with at least three nuclei as a stringent filter of multi-nucleated myotubes. The myotube coverage was defined as the area percentage of positive α-actinin signal after autothresholding using ImageJ. As a proxy for myotube width, we used an automated CellProfiler calculation to define a minor axis length based on the ellipse encompassing a mask of α-actinin that defined a single myotube. To quantify the degree of myotube alignment based on α-actinin staining, the ImageJ plugin OrientationJ was used with a Gaussian filter window of *σ* = 10 pixels to calculate the coherency and orientation angles at each pixel. Then, a global orientation order parameter was calculated from the orientation angles of at least three images per sample, as previously described (McCain et al., 2013; Petersen et al., 2020).

### Statistics

Student’s t-test was performed between week 1 and week 2 groups for each patient iDRM lines or between them, or between AO and non-treated patient iDRM lines. Comparisons with *p*-values less than 0.05 were considered statistically significant.

## Results

### Engineering Aligned DMD and LGMD2A/R1 iDRMs on Micromolded Gelatin Hydrogels

To engineer mutation-specific DMD muscle tissues *in vitro*, we isolated dermal fibroblasts from DMD 1015, DMD 1023, DMD 1003, and one control subject (1001). All fibroblasts were engineered to iDRM by expressing a tamoxifen-inducible MyoD and hTERT (Figure 1A), as described previously (Barthelemy et al., 2018; Wang et al., 2018). iDRMs were seeded on gelatin hydrogels micromolded with 10-µm-wide alternating grooves affixed to coverslips (Figure 1A). iDRMs were then triggered to become myoblasts with induction of MyoD 1–2 days after seeding and maintained in differentiation media for 1 to 2 weeks to induce fusion into myotubes (Figure 1B).

**FIGURE 2:**
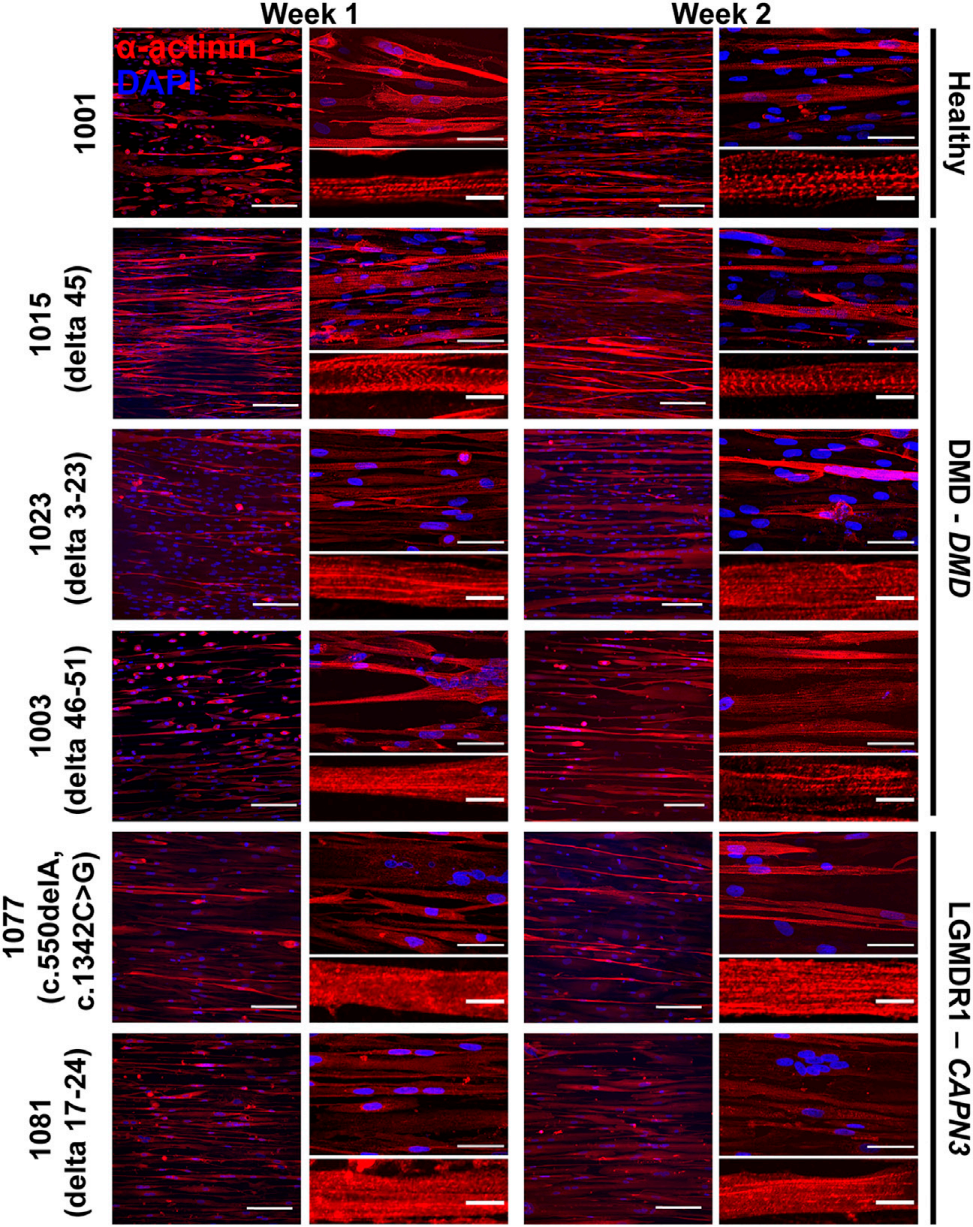
iDRMs derived from LGMD2A/R1 or DMD dermal fibroblasts on micromolded gelatin hydrogel coverslips. α-actinin staining was performed to analyze the maturation of myotubes from a healthy donor (1001) or patients presenting with LGMD2A/R1 (1077 - c.550delA, c.1342C > G and 1081 delta 17–24) or DMD (1015 – delta 45; 1023 – delta 3–23; and 1003 - delta 46–51). For each cell line, cells were differentiated for 1 or 2 weeks. Images are shown at 20× magnification on the left panel, where the scale bar represents 100 μm, and at 60× magnification in the upper right panel, where the scale bar represents 50 μm. Representative myotubes shown in the lower right panel enable visualization of sarcomeres where the scale bar represents 10 μm.

**FIGURE 3:**
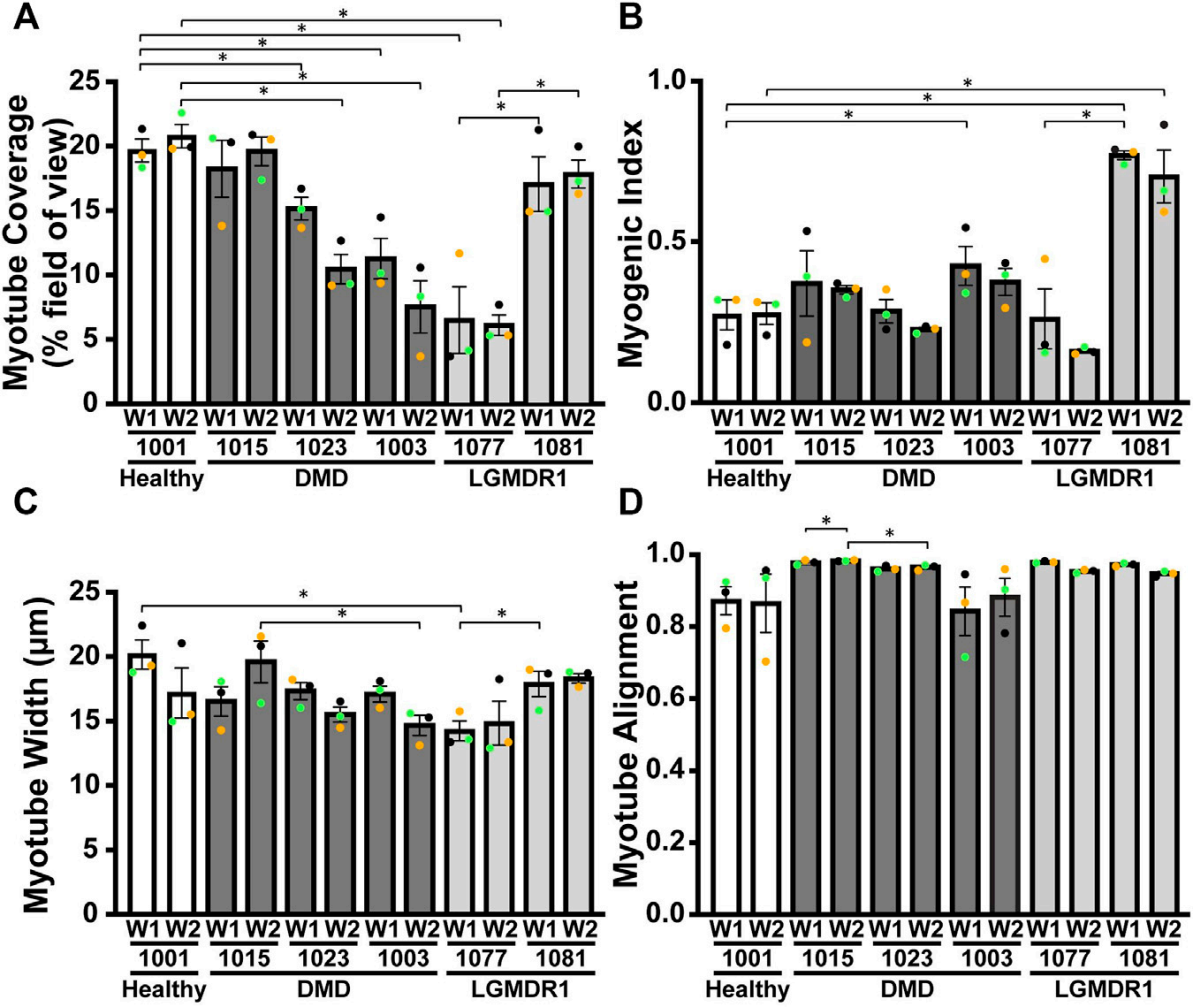
Morphological quantification of DMD or LGMD2A/R1 iDRM reveal distinct changes in mutation-specific muscle morphology. Different parameters were measured for each cell line using α-actinin staining: **(A)** α-actinin area coverage per field of view; **(B)** myogenic index representing the proportion of nuclei in myotubes (containing at least three nuclei); **(C)** minor axis length as a proxy for myotube width; **(D)** myotube alignment. Bars represent SEM. *p < 0.05; *p*-values reflect a Student’s t-test. Each experiment (*n* = 3) is represented by different color dots.

To characterize the morphological events characteristic of muscle development, we co-visualized nuclei and sarcomeric α-actinin in engineered iDRM tissues after 1 and 2 weeks in culture. We chose sarcomeric α-actinin because it is expressed relatively late in myogenesis, especially compared to other markers, such as desmin, myogenin, or myosin heavy chain (White et al., 2014; Bettadapur et al., 2016b; Nguyen et al., 2016; Denes et al., 2019b; Santoso et al., 2021b). Sarcomeric α-actinin is also present in the z-lines in sarcomeres and thus clearly demarcates mature myofibrils. All cell lines formed multi-nucleated, aligned myotubes at both time points (Figure 2), although to different degrees. To compare this, we quantified myotube coverage (α-actinin positive proportion), myogenic index (proportion of nuclei in α-actinin positive myotubes with more than three nuclei), myotube width (minor axis length), and myotube alignment (Figures 3A–D and Supplementary Figure S1). We report both myotube coverage and myogenic index to collectively indicate myotube fusion, myotube adhesion, and tissue cellularity. Healthy 1001 iDRM formed myotubes with stable sarcomeric α-actinin coverage and myogenic index throughout the 2-week culture period. Organized striations were detectable at week 1 and remained constant through week 2. Myotube width peaked in the first week and declined slightly by week 2, likely due to detachment of some of the most mature myotubes. The distribution of myotube width, shown as histograms in Supplementary Figure S1, also illustrates this trend. Myotube alignment for healthy iDRM was also high and indicated successful tissue patterning by the micromolded gelatin hydrogels.

DMD 1015 iDRM performed relatively similar to the healthy 1001 iDRM, in terms of sarcomeric α-actinin coverage (Figure 3A), myogenic index (Figure 3B), and myotube width (Figure 3C and Supplementary Figure S1), with a potential modest delay in the time to peak myotube width relative to healthy control. Similar to the healthy control, striations in DMD 1015 myotubes were also detectable (Figure 2). We attempted to quantify α-actinin striations, but the heterogeneity of the cells precluded a systematic and meaningful analysis. DMD 1023 and DMD 1003 iDRM also differentiated into multi-nucleated myotubes with near-typical myogenic index and myotube width. These results are similar to previous studies that have shown relatively unimpaired fusion in human iPSC–derived myoblasts from DMD (Caputo et al., 2020) or LGMDR9 (Ortiz-Cordero et al., 2021) patients. However, striations in DMD 1023 and DMD 1003 myotubes were qualitatively impaired relative to healthy 1001 myotubes. Likewise, myotube coverage was lower in DMD 1023 and DMD 1003 compared to healthy 1001. Myotube alignment was also high for the healthy and DMD myotubes (Figure 3D), indicating that all cell lines were instructed by the micromolded features. Myotube alignment trended lower for DMD 1003 compared to DMD 1015 and DMD 1023, possibly due to the greater severity of the mutation in this line. The healthy 1001 myotubes also had relatively low alignment, likely because myotubes tended to bridge across the micromolded features in the control line compared to the DMD lines.

To determine whether our approach was suitable for other genetic muscle diseases, we similarly isolated fibroblasts from two LGMD2A/R1 subjects, engineered them to iDRM, and differentiated them to myotubes on micromolded gelatin hydrogels (Figure 1A). LGMD2A/R1 line 1077 exhibits compound heterozygous mutations c.550delA (p.T184Rfs*36) and c.1342C > G (p448R > G) in *CAPN3*. LGMD2A/R1 line 1081 exhibits homozygous deletion of *CAPN3* exons 17 to 24 [c.(1914 + 1_1915–1)_(*544_?)del]. Whereas LGMD2A/R1 1077 showed lower α-actinin coverage (Figure 3A) and normal or lower myogenic index (Figure 3B) compared to the healthy control, LGMD2A/R1 1081 had similar sarcomere α-actinin coverage and a much higher myogenic index. For certain time points, LGMD2A/R1 1077 had lower myotube width, whereas LGMD2A/R1 1081 had higher myotube width, compared to control (Figure 3C and Supplementary Figure S1). However, neither LGMD2A/R1 1077 or LGMD2A/R1 1081 developed well-organized, discrete striations (Figure 2). Both LGMD2A/R1 1077 and LGMD2A/R1 1081 exhibited high degrees of myotube alignment (Figure 3D). Thus, our engineered tissues revealed distinct differences in disease- and mutation-specific muscle morphology, which is an advantage of using patient-derived cells. Importantly, the variability within each cell line is relatively modest.

### Morphology of Muscle Fibers in Healthy and DMD Patients

**FIGURE 4:**
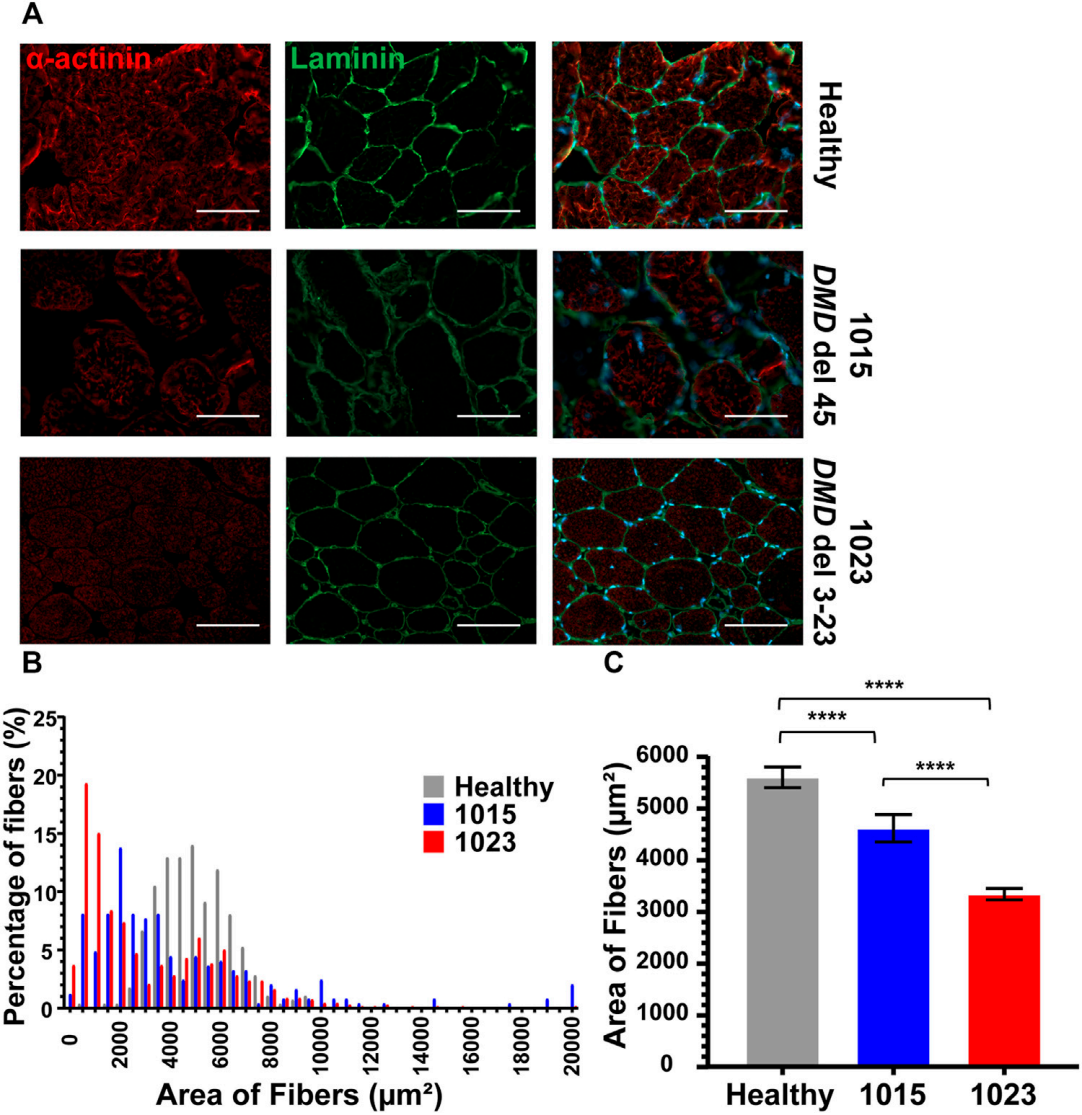
Biopsies of patients with DMD show abnormal α-actinin (1023 - delta 3–23; 1015 – delta 45). **(A)** Co-staining for α-actinin and laminin are shown. **(B)** Histogram of fiber area of muscle biopsies from healthy donor and patients presenting Duchenne muscular dystrophy sorted by 500 μm range. Number of fibers analyzed per condition was between 250 and 658. **(C)** The mean average of the fibers is also represented. Bars represent SEM. ****p < 0.0005; *p*-values reflect a Student’s t-test. Immunostains were taken at 20× magnification, where the scale bar represents 100 μm.

**FIGURE 5:**
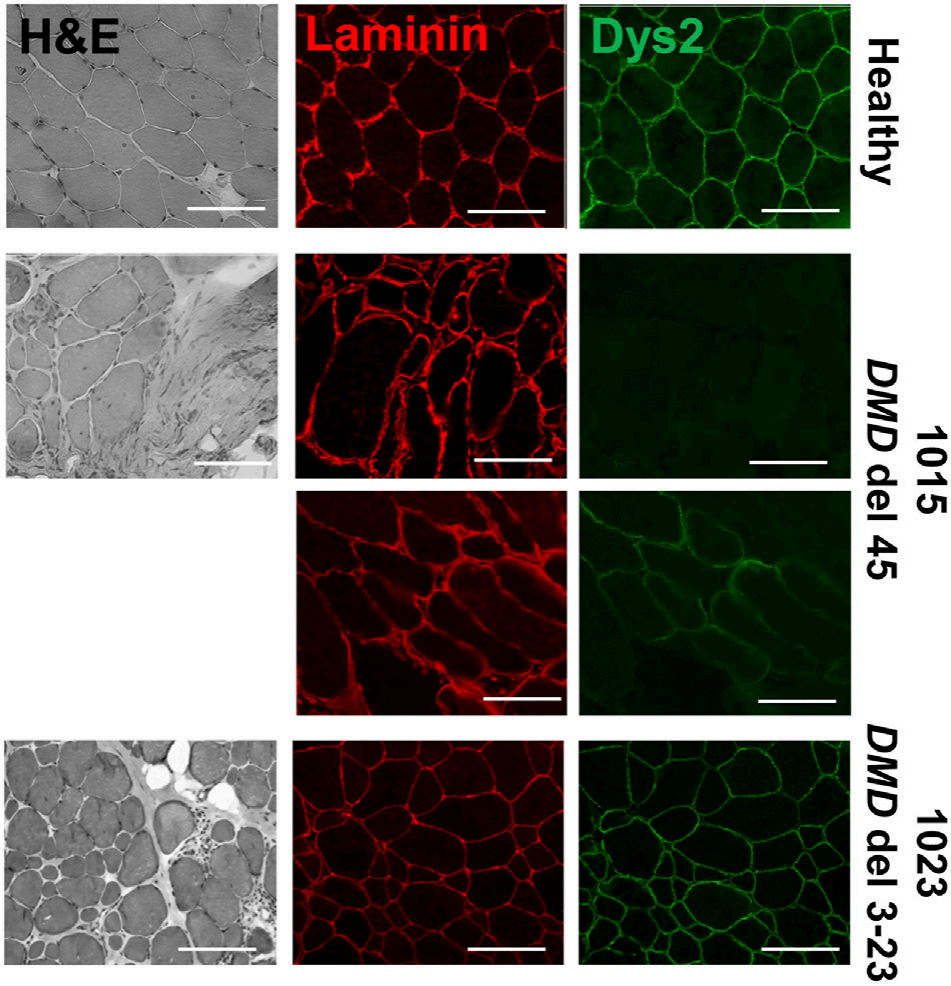
Biopsies of patients with DMD show dystrophic features. Muscle biopsy sections from patients presenting DMD (1023 - delta 3–23; 1015 – delta 45) assessed for dystrophin protein using dys2 antibody targeting the C-terminus and laminin using L9393 antibody. Hematoxylin/eosin staining for each biopsy is shown to visualize their morphological features and proceed to morphological assessment. Scale bars represent 100 μM.

As a baseline comparison for our culture models, we evaluated morphological differences in muscle biopsy tissue sections from a healthy subject and the two DMD subjects for which we had access to muscle tissue sections, DMD 1015 and DMD 1023. Both patients were biopsied before loss of ambulation and had substantial preservation of skeletal myofibers. DMD 1015 exhibits an out-of-frame deletion of *DMD* exon 45 predicted to lead to total loss of dystrophin expression. DMD 1023 exhibits an in-frame deletion of *DMD* exons 3 to 23 predicted to express a partially functional dystrophin protein lacking the actin binding site. As expected, muscle fibers in the healthy subject robustly expressed sarcomeric α-actinin throughout their cross-sectional area, were encircled by dystrophin, and were relatively consistent in size (Figure 4). Both DMD biopsies demonstrated weaker sarcomeric α-actinin staining, variations in muscle fiber size, and fibro-fatty infiltrates, concordant with a DMD phenotype (Figure 5). Sarcomeric α-actinin expression (Figure 5) and fiber size were lowest in DMD 1023 tissues despite significant expression of internally truncated sarcolemmal dystrophin protein in every myofiber (Figures 4 and 5). Whereas most 1015 fibers do not express dystrophin, we observe some patches of dystrophin expressing revertant fibers and higher α-actinin expression relative to 1023, in keeping with findings regarding α-actinin coverage in the iDRM culture models (Figures 2 and 3). However, it is important to consider that the degeneration process *in vivo* is vastly more complex and on a much longer timescale than what can be observed *in vitro*. Thus, comparing patient muscle biopsies to *in vitro* engineered muscle tissues should be done with much caution and conservativeness (Forbes et al., 2020).

### Evaluation of Dystrophin Rescue in DMD iDRMs Treated With Exon Skipping AOs

Development of precision medicines requires human mutation specific platforms for assessing mechanisms of action and efficacy. AO DMD exon skipping drugs function to reframe mutant *DMD* mRNA through removal of an “extra” exon, enabling rescue of an internally deleted but partially functional dystrophin protein. “Exon skipping” drugs are mutation-specific, in so far as only some *DMD* mutations adjacent to the targeted region can be reframed. 1015 mutation can be rendered in frame by exclusion of exon 44, whereas 1003 mutation is amenable to reframing by targeting exon 45.

**FIGURE 6:**
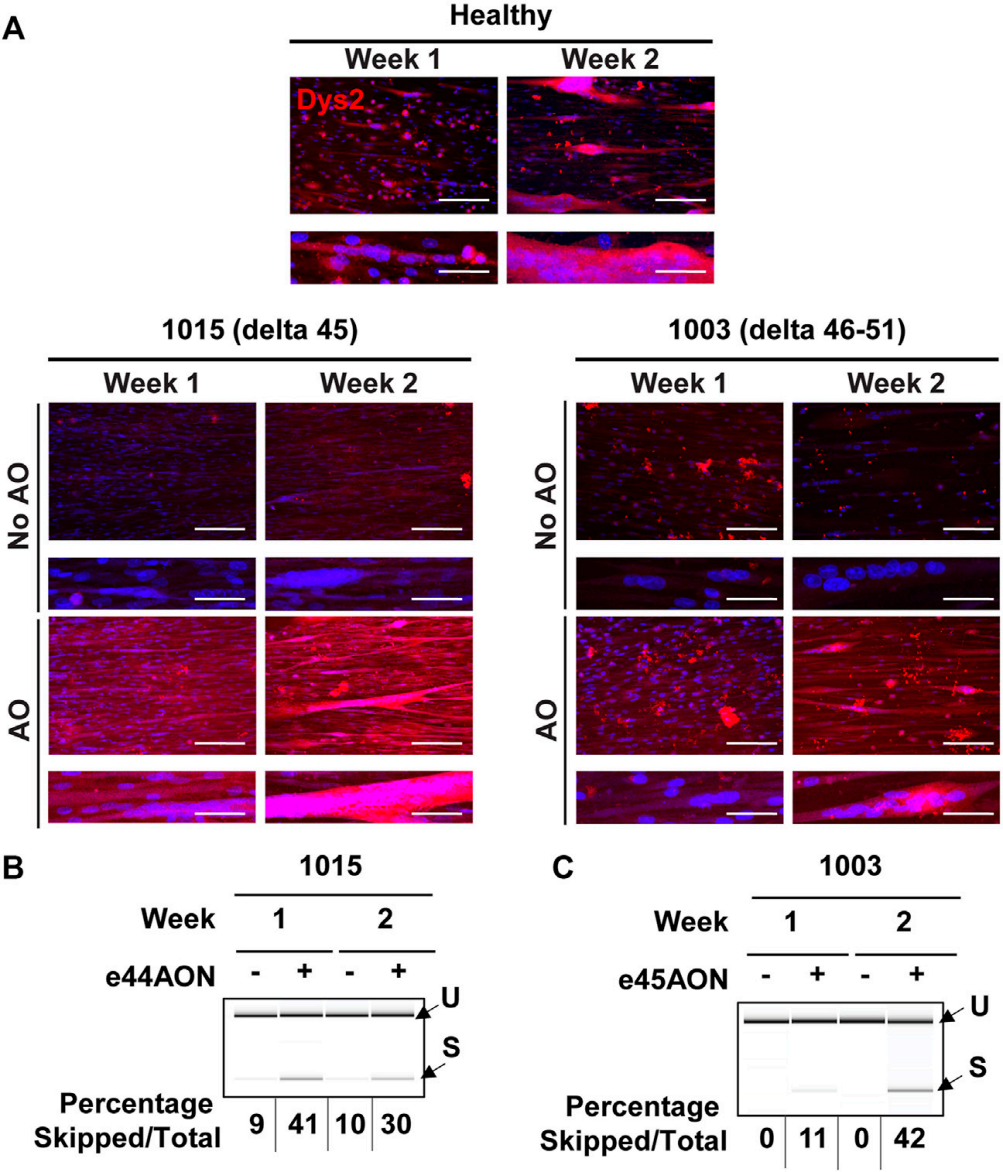
Dystrophin rescue by *DMD* exon skipping in DMD 1015 and DMD 1003 iDRMs. DMD 1015 (delta 45) and DMD 1003 (delta 46–51) iDRMs were cultured for 1 or 2 weeks before the addition of antisense oligonucleotides and, 2 days later, immunostaining or RNA extraction (on pooled triplicate) were performed. **(A)** Dystrophin expression in healthy and DMD iDRM tissues was visualized using Dys2 antibody (c-ter). Images are shown at 20x magnification in the top panel, where the scale bar represents 100 μm, and at 60x magnification in the lower panel to enable visualization of sarcomeres, where the scale bar represents 30 μm. After RT-PCR, samples were run on chips to analyze exon skipping. The percentage of skipped mRNA over the total (skipped plus unskipped mRNA) is indicated for **(B)** DMD 1015 and **(C)** DMD 1003.

To determine whether AO treatment rescued dystrophin expression in iDRM, DMD 1015 and DMD 1003 iDRMs were exposed to exon skipping drug targeting exon 44 or exon 45, respectively, after 1 or 2 weeks of differentiation on micromolded hydrogels. We performed immunostaining on healthy and DMD iDRM engineered tissues treated with or without AO to determine whether we could observe rescue of dystrophin (Figure 6A). Myotubes generated from the healthy iDRM demonstrated dystrophin staining at both time points (Figure 6A). In DMD 1015 iDRMs, low levels of dystrophin protein were detected without AO treatment after 2 week of culture on the micromolded platform, consistent with detection of low levels of skipped DMD message in the absence of AO. Exposure to AO increased the rescued exon 44 deleted DMD mRNA and dystrophin proteins expression and after both 1 and 2 weeks of culture, consistent with levels of exon 45 skipped mRNA induced (Figure 6B). In DMD 1003 iDRMs, dystrophin was not detectable in 1003 in the absence of skipping drug. Exposure to exon 45 skipping AO rescued low levels of dystrophin protein when AO was added after 1 week and to a greater extent when AO was added after 2 weeks of differentiation (Figures 6A,B). Thus, both DMD 1015 and DMD 1003 demonstrated brighter dystrophin staining with the addition of skipping AO.

**FIGURE 7:**
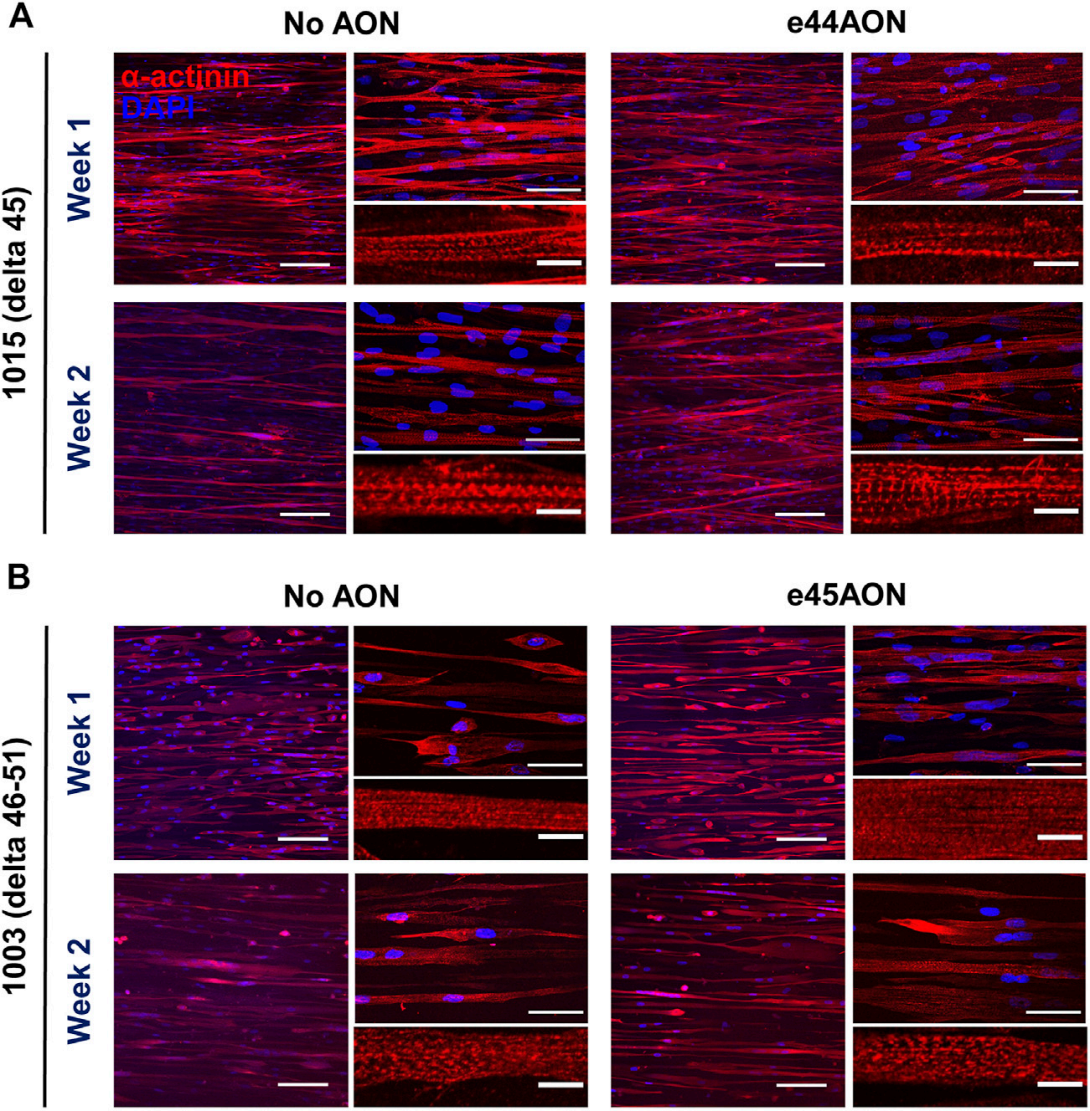
Effects of dystrophin rescue by exon skipping on α-actinin expression and organization in. DMD 1015 (delta 45) or DMD 1003 (delta 46–51). **(A)** DMD 1015 or **(B)** DMD 1003 iDRMs were cultured for 1 or 2 weeks before the addition of antisense oligonucleotides. α-actinin staining were performed to analyze the maturation of myotubes. Images are shown at 20× magnification in the left panel, where the scale bar represents 100 μm, and at 60× magnification in the upper right panel, where the scale bar represents 50 μm. Representative myotubes shown in the lower right panel enable visualization of sarcomeres, where the scale bar represents 10 μm.

**FIGURE 8:**
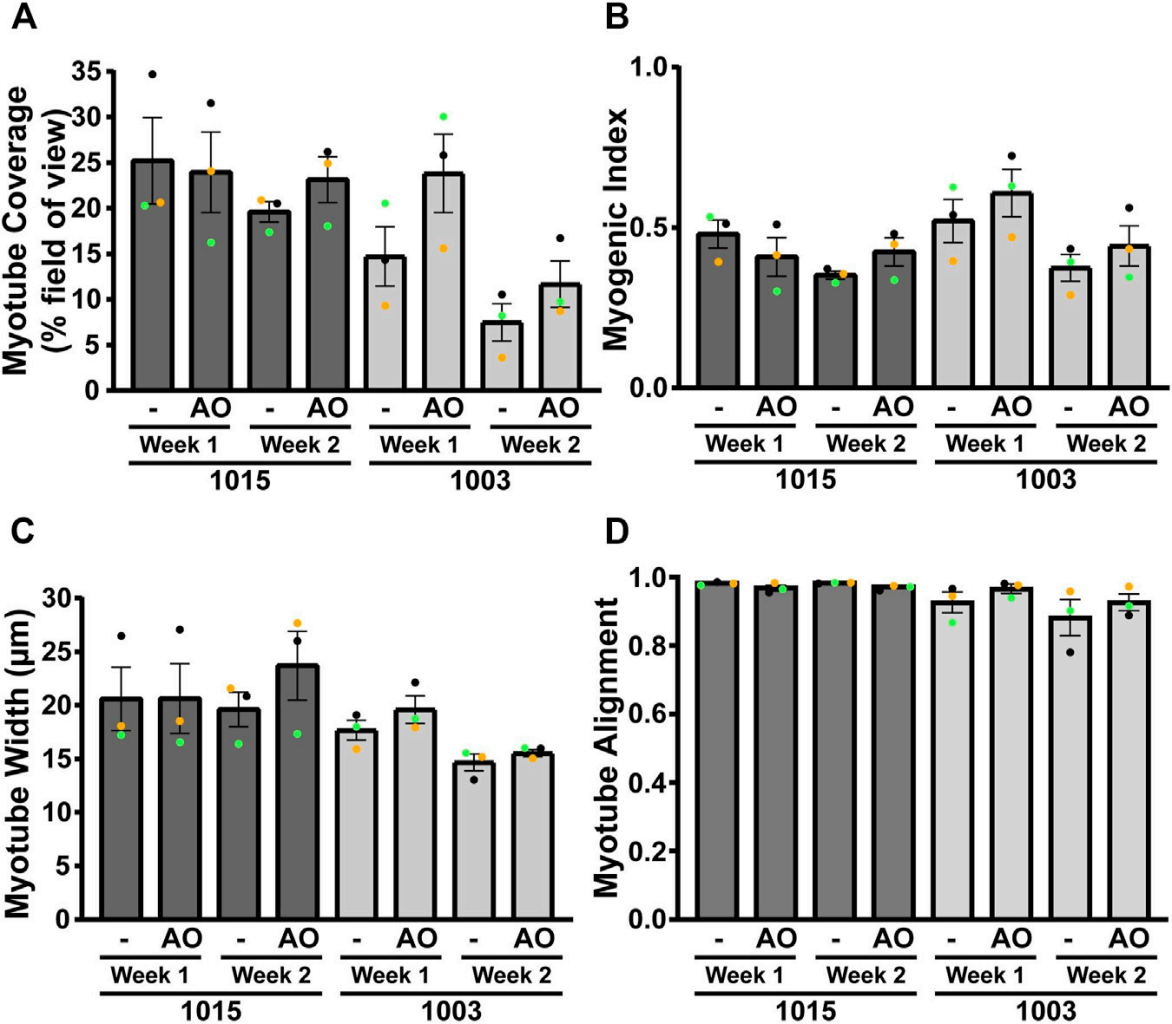
Morphological quantification of AO-treated DMD 1015 and DMD 1003 iDRMs. Different parameters were measured for each cell line using α-actinin staining: **(A)** α-actinin area coverage per field of view; **(B)** myogenic index representing the proportion of nuclei in myotubes (containing at least three nuclei); **(C)** minor axis length as a proxy for myotube width; and **(D)** myotube alignment. Bars represent SEM. Each experiment (*n* = 3) is represented by different color dots.

We next measured the effects of dystrophin rescue induced by exon skipping on myotube morphology for DMD 1015 and DMD1003 at week 1 or 2 of differentiation (Figure 7). We performed staining for α-actinin and nuclei (Figure 7) and calculated myotube coverage (Figure 8A), myogenic index, (Figure 8B), myotube width (Figure 8C and Supplementary Figure S2), and myotube alignment (Figure 8D). DMD 1015 iDRMs developed striations at weeks 1 and 2, regardless of exon skipping and dystrophin rescue. There were also no noticeable changes in myogenic index, myotube coverage, myotube width, or myotube alignment in 1015 due to AO treatment, except for slight but non-significant increases in the first three metrics after 2 weeks of AO treatment. The distribution of myotube width from iDRM DMD 1015 and 1003 samples with and without AO treatment is shown as histograms in Supplementary Figure S2 and shows a particular shift toward a higher proportion of wider myotubes after AO treatment at week 2. The effects on dystrophin rescue in DMD 1003 were more noticeable, with exon skipping AO inducing greater myotube coverage, myogenic index, and myotube alignment at both time points. However, these increases did not reach statistical significance. Nonetheless, taken together, these findings highlight a potential role for rescued dystrophin protein in maturation or stability of myotubes and sarcomeres.

## Discussion

Development of genetic medicines aimed at repairing or replacing DMD and LGMDDR1/LGMD2A mutations is an active area of research, and several drugs have been already approved or are in human pre-clinical or clinical trials (Sheikh and Yokota, 2021). However, there is a paucity of DMD and LGMD mutation specific human culture models for assessing mechanisms of action or improving efficacy of therapeutic strategies. Here, we report that culturing one heathy subject, three DMD, and two LGMD-derived iDRM on micromolded gelatin hydrogel coverslips induces tissue alignment, prolongs culture lifetime, and promotes myotube development in culture over 2 weeks. This relatively long culture period enabled us to characterize myotube development and dystrophin rescue in DMD myotubes in response to AO skipping drugs. We also show that our approach is compatible with iDRM from LGMD2A/R1 patients, demonstrating its modularity for modeling other forms of muscular dystrophy.

Although all healthy and DMD iDRM demonstrated myogenic potential and a high degree of alignment, DMD 1003 showed the most severe defects in α-actinin expression, followed by DMD 1023. We were largely unable to distinguish DMD 1015 from healthy control using these measures. The *in vitro* results for DMD 1015 and DMD 1023 mirror the trends of the patient muscle biopsies, for which defects were more substantial for DMD 1023 compared to DMD 1015. Thus, our combination of patient-derived iDRM with micromolded gelatin hydrogels replicated select histological phenotypes of native muscle and further demonstrates that muscle development *in vitro* is regulated by distinct *DMD* mutations.

iDRM DMD 1003 harbors a deletion of exons 46–51 of *DMD*, which encodes an out-of-frame mRNA, produces no dystrophin protein, and demonstrates defects in α-actinin coverage and the development of striations. These data support a role for dystrophin in the development or stabilization of myotubes and sarcomeres. Such a suggestion is consistent with reports that the dystrophin-glycoprotein complex slows depolymerization of actin filaments *in vitro* (Rybakova et al., 1996; Rybakova and Ervasti, 1997). Alternatively, low α-actinin coverage and myotube width may be secondary to impaired cell survival or earlier developmental defects dependent on dystrophin. *DMD* reframing and dystrophin rescue induced within DMD 1003 by exposure to exon 45 skipping AO modestly improved measures of myotube coverage, fusion, and alignment, further supporting a role for dystrophin in these processes and highlighting the value of this model as a screening tool for drug development and optimization of precision medicines.

iDRM DMD 1023 has an in-frame mutation *DMD* 3–23, which creates substantial amounts of dystrophin properly localized at the sarcolemma but lacking the primary actin binding site (Gibbs et al., 2019). We identified defects in myotube maturation and organization, potentially highlighting requirements for dystrophin actin binding in myogenesis and myotube sarcomere maturation, consistent with a previous report (Banks et al., 2007). Additional experiments will be needed to support this hypothesis.

iDRM DMD 1015 has an exon 45 deletion of *DMD* amenable to reframing by exon 44 AO skipping. We have previously demonstrated that iDRM DMD 1015 constitutively expresses low levels of exon 45 skipped and reframed *DMD* mRNA, even in the absence of AO, as we have reported for several DMD iDRM with exon 45 deletions (Wang et al., 2018). Likewise, immunostaining frozen muscle biopsy sections from the DMD 1015 fibroblast donor showed some clusters expressing low levels of dystrophin rescue *in vivo*. The predisposition of exon 45 deletion *DMD* mutants to self-correct by skipping exon 44 to produce low levels of dystrophin has been suggested as the molecular basis of mild disease progression relative to typical *DMD* frameshifting mutations and highlights that even low levels of dystrophin can have functional consequences. We also detected low levels of exon 45 skipped *DMD* mRNA in the absence of any treatment and increased exon 44 exclusion and robust induction of dystrophin protein expression in cells exposed to skipping AO. Unlike the other two DMD patient-derived iDRM, DMD 1015 behaved much closer to wild type, with no defect in α-actinin coverage or striation. It is possible that the low levels of rescued dystrophin constitutively expressed in DMD 1015 are sufficient to partially overcome the developmental defects in myotube development or sarcomere stability observed in dystrophin null DMD 1003. Exon 44 skipping AO increased dystrophin expression and induced modest improvements in myogenic index, myotube coverage, and myotube width, further supporting a role for rescued dystrophin in facilitation of myotube maturation in DMD 1015. Moreover, multiple α-actinin isoforms have been linked to dystrophin and DMD. α-Actinin 3 has been identified as a known modifier of DMD, α-actinin 2 has been identified as an extended member of the DGC, and a progressive depletion of α-actinin proteins has been observed in DMD (Minetti et al., 1991; Hance et al., 1999; Hogarth et al., 2017), making this protein family an underestimated marker of pathophysiological changes in this disease.

Although differentiation on micromolded gelatin hydrogels enabled visualization of rescued dystrophin expression, we did not observe the expected patterning of dystrophin. In native muscle, dystrophin is enriched in costamere protein assemblies, which circumferentially align with the α-actinin enriched Z disk and couple force-generating sarcomeres with the sarcolemma. Similarly, although we did detect some punctate sarcomere-like structures in myotubes derived from select iDRM lines, the overall maturity of the myofibrils and sarcomeres was limited, especially compared to myotubes derived from primary myoblasts. Similar issues related to myofibril immaturity have routinely been observed in myotubes derived from a variety of reprogrammed (Boularaoui et al., 2018) and iPSC-derived myoblasts (Lainé et al., 2018; Rao et al., 2018) and likely reduce the baseline and drug-induced differences in the phenotypes of healthy and diseased cells. Overall, the maturity of iDRM-derived myotubes observed in this study was similar or weaker than iPSC-derived myotubes (Caputo et al., 2020; Ortiz-Cordero et al., 2021). Of note, however, iDRMs require less time, cost, and expertise to generate compared to iPSC-derived myoblasts, which may be especially beneficial for generating patient-specific muscle tissues in time-sensitive or resource-limited settings. Extending culture time (Santoso and McCain, 2021), integrating supporting cell types (Juhas et al., 2018; Santosa et al., 2018; Santoso and McCain, 2021), providing electrical (Nedachi et al., 2008; Chen et al., 2021) or mechanical stimulation (Heher et al., 2015; Chang et al., 2016), or engineering 3-D tissues (Madden et al., 2015; Uzel et al., 2016; Costantini et al., 2017; Davis et al., 2019; Ariyasinghe et al., 2020; Ebrahimi et al., 2021) or earlier exposure to AO could also help induce muscle maturation and proper localization of dystrophin and α-actinin.

LGMD2A/R1 1077 express mutations [c.550delA (p.T184Rfs*36) and c.1342C > G (p448R > G] within the catalytic PC1 and the calcium-binding and phospholipid-binding C2 domains, respectively, and these iDRMs show defects in formation of myotube maturation and structure. Patient cells derived from patient LGMD2A/R1 1081, present with a homozygous deletion of the entire c-terminal region of the protein, were able to form myotubes and showed no quantitative defects in α-actinin coverage or axis length but did not develop proper maturation of the clearly defined α-actinin marked sarcomeres upon visual inspection. It is unclear why LGMD 1077 iDRM demonstrates significant defects in myotube maturation, whereas 1081 has an exceptionally high MI and is otherwise near normal.

One advantage of differentiating myotubes in culture is that it allows assessment of sequential myoblast activation, fusion, and the development of mature myofibers with organized sarcomeres, responsible for striated muscle patterning and required for force generation. Thus, iDRM differentiated on micromolded hydrogels may aid in dissecting requirements for each of these developmental events (White et al., 2014). However, factors other than DMD or LGMD mutation that might influence iDRM performance include the presence/absence of DMD or LGMD2A/R1 disease modifier genes or artifacts of culture selection. Therefore, comparisons between individually derived iDRM to determine relative myogenic activity of particular mutations should be made with caution. Rather, creation of isogenic iDRM expressing defined DMD or LGMD mutations and full-length dystrophin or a panel of distinct patient iDRM with similar mutations may be necessary to confirm preliminary observations made regarding effects of DMD or LGMD mutation based on comparison between two lines. Alternatively, experiments where iDRM DMD 1003 and 1015 activity is measured before and after exposure to a potential therapeutic or disease modifying entity, such as exon skipping AO, do not suffer from this criticism and thus are likely to prove valuable for precision drug optimization and screening.

In summary, our results establish that patient-derived iDRM differentiate into aligned myotubes on micromolded gelatin hydrogels and enable evaluation of myotube formation as a function of patient-specific mutations. This is a promising approach for screening and testing personalized therapies for muscular dystrophies and other genetic muscle diseases *in vitro*. Together, our findings highlight the benefit of human cell models combined with engineered scaffolds that more closely mimic the *in vivo* microenvironment and encourage the development of new strategies to promote further maturation for proper characterization of morphological differences and evaluation of the efficacy of new therapies.

## Data Availability

The raw data supporting the conclusion of this article will be made available by the authors, without undue reservation.

## Supplementary Material

Supplementary Figure S1 Dystrophic myotubes showed a delayed myotubes enlargement at week 1 that fades away at week 2. α-Actinin staining were performed to analyze the maturation of myotubes from a healthy donor (1001) or patients presenting with LGMD2A/R1 (1077 - c.550delA, c.1342C > G and 1081 - delta 17–24) or DMD (1015 - delta 45; 1023 - delta 3–23; and 1003 - delta 46–51). For each cell line, cells were differentiated for 1 or 2 weeks and all the myotubes showing more than three nuclei from two independent experiments run in duplicates were analyzed and compiled on these graphs representing the width ranges (in µm) observed in our cell culture models.

Supplementary Figure S2 AO treatment promotes enlargement of myotubes from DMD patients’ cell lines. DMD 1015 (delta 45) or DMD 1003 (delta 46–51) cells were cultured for 1 or 2 weeks before the addition of antisense oligonucleotides and α-actinin staining were performed to analyze the maturation of myotubes. All the myotubes showing more than three nuclei from three independent experiments run in duplicates were analyzed and compiled on these graphs representing the width ranges (in µm) observed in our cell culture models.

## References

[B1] Al Tanoury Z. Zimmerman J. F. Rao J. Sieiro D. McNamara H. M. Cherrier T. (2021). Prednisolone Rescues Duchenne Muscular Dystrophy Phenotypes in Human Pluripotent Stem Cell-Derived Skeletal Muscle *In Vitro* . Proc. Natl. Acad. Sci. USA 118 (28), e2022960118. 10.1073/pnas.2022960118 34260377 PMC8285911

[B2] Ariyasinghe N. R. Santoso J. W. Gupta D. Pincus M. J. August P. R. McCain M. L. (2020). Optical Clearing of Skeletal Muscle Bundles Engineered in 3-D Printed Templates. Ann. Biomed. Eng. 49, 523–535. 10.1007/s10439-020-02583-0 32748107

[B3] Banks G. B. Gregorevic P. Allen J. M. Finn E. E. Chamberlain J. S. (2007). Functional Capacity of Dystrophins Carrying Deletions in the N-Terminal Actin-Binding Domain. Hum. Mol. Genet. 16 (17), 2105–2113. 10.1093/hmg/ddm158 17588958

[B4] Barthélémy F. Courrier S. Lévy N. Krahn M. Bartoli M. (2018). Dysferlin Exon 32 Skipping in Patient Cells. Methods Mol. Biol. 1828, 489–496. 10.1007/978-1-4939-8651-4_31 30171562

[B5] Barthelemy F. Wang D. Nelson S. F. Miceli M. C. (2018). Validation and Detection of Exon Skipping Boosters in DMD Patient Cell Models and Mdx Mouse. Methods Mol. Biol. 1828, 309–326. 10.1007/978-1-4939-8651-4_19 30171550

[B6] Barthélémy F. Wang R. T. Hsu C. Douine E. D. Marcantonio E. E. Nelson S. F. (2019). Targeting RyR Activity Boosts Antisense Exon 44 and 45 Skipping in Human DMD Skeletal or Cardiac Muscle Culture Models. Mol. Ther. - Nucleic Acids 18, 580–589. 10.1016/j.omtn.2019.09.020 31678734 PMC6838898

[B7] Barthelemy F. Woods J. D. Nieves‐Rodriguez S. Douine E. D. Wang R. Wanagat J. (2020). A Well‐tolerated Core Needle Muscle Biopsy Process Suitable for Children and Adults. Muscle & Nerve 62 (6), 688–698. 10.1002/mus.27041 32820569 PMC7756388

[B8] Bettadapur A. Suh G. C. Geisse N. A. Wang E. R. Hua C. Huber H. A. (2016). Prolonged Culture of Aligned Skeletal Myotubes on Micromolded Gelatin Hydrogels. Sci. Rep. 6, 28855. 10.1038/srep28855 27350122 PMC4924097

[B9] Bettadapur A. Suh G. C. Geisse N. A. Wang E. R. Hua C. Huber H. A. (2016). Prolonged Culture of Aligned Skeletal Myotubes on Micromolded Gelatin Hydrogels. Sci. Rep. 6, 28855. 10.1038/srep28855 27350122 PMC4924097

[B10] BhattBHATT1 A. D. K. S. Shah K. Puvar A. Joshi C. G. Joshi M. (2019). A Case of Limb Girdle Muscular Dystrophy Type 2A from India: Copy Number Variation Analysis Using Targeted Amplicon Sequencing. Jcdr 13 12812. 10.7860/JCDR/2019/40923.12812

[B11] Blau H. M. Webster C. Pavlath G. K. (1983). Defective Myoblasts Identified in Duchenne Muscular Dystrophy. Proc. Natl. Acad. Sci. 80 (15), 4856–4860. 10.1073/pnas.80.15.4856 6576361 PMC384144

[B12] Boularaoui S. M. Abdel‐Raouf K. M. A. Alwahab N. S. A. Kondash M. E. Truskey G. A. Teo J. C. M. (2018). Efficient Transdifferentiation of Human Dermal Fibroblasts into Skeletal Muscle. J. Tissue Eng. Regen. Med. 12 (2), e918–e36. 10.1002/term.2415 28101909

[B13] Caputo L. Granados A. Lenzi J. Rosa A. Ait-Si-Ali S. Puri P. L. (2020). Acute Conversion of Patient-Derived Duchenne Muscular Dystrophy iPSC into Myotubes Reveals Constitutive and Inducible Over-activation of TGFβ-dependent Pro-fibrotic Signaling. Skeletal Muscle 10 (1), 13. 10.1186/s13395-020-00224-7 32359374 PMC7195779

[B14] Chang Y.-J. Chen Y.-J. Huang C.-W. Fan S.-C. Huang B.-M. Chang W.-T. (2016). Cyclic Stretch Facilitates Myogenesis in C2C12 Myoblasts and Rescues Thiazolidinedione-Inhibited Myotube Formation. Front. Bioeng. Biotechnol. 4, 27. 10.3389/fbioe.2016.00027 27047938 PMC4800178

[B15] Chaouch S. Mouly V. Goyenvalle A. Vulin A. Mamchaoui K. Negroni E. (2009). Immortalized Skin Fibroblasts Expressing Conditional MyoD as a Renewable and Reliable Source of Converted Human Muscle Cells to Assess Therapeutic Strategies for Muscular Dystrophies: Validation of an Exon-Skipping Approach to Restore Dystrophin in Duchenne Muscular Dystrophy Cells. Hum. Gene Ther. 20 (7), 784–790. 10.1089/hum.2008.163 19358679

[B16] Chen Z. Li B. Zhan R.-Z. Rao L. Bursac N. (2021). Exercise Mimetics and JAK Inhibition Attenuate IFN-γ-Induced Wasting in Engineered Human Skeletal Muscle. Sci. Adv. 7 9502. 10.1126/sciadv.abd9502 PMC10964957 33523949

[B17] Costantini M. Testa S. Fornetti E. Barbetta A. Trombetta M. Cannata S. M. (2017). Engineering Muscle Networks in 3D Gelatin Methacryloyl Hydrogels: Influence of Mechanical Stiffness and Geometrical Confinement. Front. Bioeng. Biotechnol. 5, 22. 10.3389/fbioe.2017.00022 28439516 PMC5383707

[B18] Davis B. N. J. Santoso J. W. Walker M. J. Oliver C. E. Cunningham M. M. Boehm C. A. (2019). Modeling the Effect of TNF-α upon Drug-Induced Toxicity in Human, Tissue-Engineered Myobundles. Ann. Biomed. Eng. 47, 1596–1610. 10.1007/s10439-019-02263-8 30963383 PMC6559943

[B19] Denes L. T. Riley L. A. Mijares J. R. Arboleda J. D. McKee K. Esser K. A. (2019). Culturing C2C12 Myotubes on Micromolded Gelatin Hydrogels Accelerates Myotube Maturation. Skeletal Muscle 9 (1), 17. 10.1186/s13395-019-0203-4 31174599 PMC6555731

[B20] Denes L. T. Riley L. A. Mijares J. R. Arboleda J. D. McKee K. Esser K. A. (2019). Culturing C2C12 Myotubes on Micromolded Gelatin Hydrogels Accelerates Myotube Maturation. Skeletal Muscle 9 (1), 17. 10.1186/s13395-019-0203-4 31174599 PMC6555731

[B21] Flanigan K. M. (2014). Duchenne and Becker Muscular Dystrophies. Neurol. Clin. 32 (3), 671–688. 10.1016/j.ncl.2014.05.002 25037084

[B22] Forbes S. C. Arora H. Willcocks R. J. Triplett W. T. Rooney W. D. Barnard A. M. (2020). Upper and Lower Extremities in Duchenne Muscular Dystrophy Evaluated with Quantitative MRI and Proton MR Spectroscopy in a Multicenter Cohort. Radiology 295 (3), 616–625. 10.1148/radiol.2020192210 32286193 PMC7263287

[B23] Gibbs E. M. Barthélémy F. Douine E. D. Hardiman N. C. Shieh P. B. Khanlou N. (2019). Large In-Frame 5′ Deletions in DMD Associated with Mild Duchenne Muscular Dystrophy: Two Case Reports and a Review of the Literature. Neuromuscul. Disord. 29 (11), 863–873. 10.1016/j.nmd.2019.09.009 31672265 PMC7092699

[B24] Gupta D. Santoso J. W. McCain M. L. (2021). Characterization of Gelatin Hydrogels Cross-Linked with Microbial Transglutaminase as Engineered Skeletal Muscle Substrates. Bioengineering 8 (1), 6. 10.3390/bioengineering8010006 33418892 PMC7825108

[B25] Hance J. E. Fu S. Y. Watkins S. C. Beggs A. H. Michalak M. (1999). α-Actinin-2 Is a New Component of the Dystrophin-Glycoprotein Complex. Arch. Biochem. Biophys. 365 (2), 216–222. 10.1006/abbi.1999.1172 10328815

[B26] Heher P. Maleiner B. Prüller J. Teuschl A. H. Kollmitzer J. Monforte X. (2015). A Novel Bioreactor for the Generation of Highly Aligned 3D Skeletal Muscle-like Constructs through Orientation of Fibrin via Application of Static Strain. Acta Biomater. 24, 251–265. 10.1016/j.actbio.2015.06.033 26141153

[B27] Hogarth M. W. Houweling P. J. Houweling P. J. Thomas K. C. Gordish-Dressman H. Bello L. (2017). Evidence for ACTN3 as a Genetic Modifier of Duchenne Muscular Dystrophy. Nat. Commun., 8, 14143. 10.1038/ncomms14143 28139640 PMC5290331

[B28] Juhas M. Abutaleb N. Wang J. T. Ye J. Shaikh Z. Sriworarat C. (2018). Incorporation of Macrophages into Engineered Skeletal Muscle Enables Enhanced Muscle Regeneration. Nat. Biomed. Eng. 2, 942–954. 10.1038/s41551-018-0290-2 30581652 PMC6296488

[B29] Kendall G. C. Mokhonova E. I. Moran M. Sejbuk N. E. Wang D. W. Silva O. (2012). Dantrolene Enhances Antisense-Mediated Exon Skipping in Human and Mouse Models of Duchenne Muscular Dystrophy. Sci. Transl. Med. 4 (164), 164ra0. 10.1126/scitranslmed.3005054 23241744

[B30] Kim E. Y. Page P. Dellefave-Castillo L. M. McNally E. M. Wyatt E. J. (2016). Direct Reprogramming of Urine-Derived Cells with Inducible MyoD for Modeling Human Muscle Disease. Skeletal Muscle 6, 32. 10.1186/s13395-016-0103-9 27651888 PMC5025576

[B31] Krahn M. Bernard R. Pécheux C. Hammouda E. H. Eymard B. Munain A. L. d. (2006). Screening of the CAPN3 Gene in Patients with Possible LGMD2A. Clin. Genet. 69 (5), 444–449. 10.1111/j.1399-0004.2006.00603.x 16650086

[B32] Kramerova I. Beckmann J. S. Spencer M. J. (2007). Molecular and Cellular Basis of Calpainopathy (Limb Girdle Muscular Dystrophy Type 2A). Biochim. Biophys. Acta (Bba) - Mol. Basis Dis. 1772 (2), 128–144. 10.1016/j.bbadis.2006.07.002 16934440

[B33] Lainé J. Skoglund G. Fournier E. Tabti N. (2018). Development of the Excitation-Contraction Coupling Machinery and its Relation to Myofibrillogenesis in Human iPSC-Derived Skeletal Myocytes. Skeletal Muscle 8 (1), 1. 10.1186/s13395-017-0147-5 29304851 PMC5756430

[B34] Lee C. C. Hoang A. Segovia D. Herbst A. Barthelemy F. Gibbs E. (2020). Enhanced Methods for Needle Biopsy and Cryopreservation of Skeletal Muscle in Older Adults. jch 11 553. 10.37421/jch.2020.11.553 PMC7304549 32566369

[B35] Madden L. Juhas M. Kraus W. E. Truskey G. A. Bursac N. (2015). Bioengineered Human Myobundles Mimic Clinical Responses of Skeletal Muscle to Drugs. eLife 4 4885. 10.7554/eLife.04885 PMC4337710 25575180

[B36] Maffioletti S. M. Sarcar S. Henderson A. B. H. Mannhardt I. Pinton L. Moyle L. A. (2018). Three-Dimensional Human iPSC-Derived Artificial Skeletal Muscles Model Muscular Dystrophies and Enable Multilineage Tissue Engineering. Cel Rep. 23 (3), 899–908. 10.1016/j.celrep.2018.03.091 PMC5917451 29669293

[B37] McCain M. L. Sheehy S. P. Grosberg A. Goss J. A. Parker K. K. (2013). Recapitulating Maladaptive, Multiscale Remodeling of Failing Myocardium on a Chip. Proc. Natl. Acad. Sci. 110 (24), 9770–9775. 10.1073/pnas.1304913110 23716679 PMC3683774

[B38] Ebrahimi M. Fusto H. Tiper A. Datye Y. Christine A. Nguyen C. T. (2021). De Novo revertant Fiber Formation and Therapy Testing in a 3D Culture Model of Duchenne Muscular Dystrophy Skeletal Muscle. Acta Biomater. 132, 227–244. 10.1016/j.actbio.2021.05.020 34048976

[B39] Minetti C. Ricci E. Bonilla E. (1991). Progressive Depletion of Fast Alpha-Actinin-Positive Muscle Fibers in Duchenne Muscular Dystrophy. Neurology 41 (12), 1977. 10.1212/wnl.41.12.1977 1745358

[B40] Muntoni F. (2001). Is a Muscle Biopsy in Duchenne Dystrophy Really Necessary? Neurology 57 (4), 574–575. 10.1212/wnl.57.4.574 11524463

[B41] Nallamilli B. R. R. Chakravorty S. Kesari A. Tanner A. Ankala A. Schneider T. (2018). Genetic Landscape and Novel Disease Mechanisms from a largeLGMDcohort of 4656 Patients. Ann. Clin. Transl Neurol. 5 (12), 1574–1587. 10.1002/acn3.649 30564623 PMC6292381

[B42] Nedachi T. Fujita H. Kanzaki M. (2008). Contractile C2C12myotube Model for Studying Exercise-Inducible Responses in Skeletal Muscle. Am. J. Physiology-Endocrinology Metab. 295 (5), E1191–E1204. 10.1152/ajpendo.90280.2008 18780777

[B43] Nesmith A. P. Wagner M. A. Pasqualini F. S. O’Connor B. B. Pincus M. J. August P. R. (2016). A Human *In Vitro* Model of Duchenne Muscular Dystrophy Muscle Formation and Contractility. J. Cel. Biol. 215 (1), 47–56. 10.1083/jcb.201603111 PMC5057287 27697929

[B44] Nguyen N.-U. -N. Liu T.-Y. Wang H.-V. (2016). Timing Appearance and Integration of Actin-Organizing Palladin Protein in Dynamic Myofibril Assembly. bioRxiv, 047183. 10.1101/047183

[B45] Ortiz-Cordero C. Bincoletto C. Dhoke N. R. Selvaraj S. Magli A. Zhou H. (2021). Defective Autophagy and Increased Apoptosis Contribute toward the Pathogenesis of FKRP-Associated Muscular Dystrophies. Stem Cel Rep. 16 (11), 2752–2767. 10.1016/j.stemcr.2021.09.009 PMC8581053 34653404

[B46] Pakula A. Spinazzola J. M. Gussoni E. (2019). Purification of Myogenic Progenitors from Human Muscle Using Fluorescence-Activated Cell Sorting (FACS). Methods Mol. Biol. 1889, 1–15. 10.1007/978-1-4939-8897-6_1 30367405 PMC6472699

[B47] Petersen A. P. Cho N. Lyra-Leite D. M. Santoso J. W. Gupta D. Ariyasinghe N. R. (2020). Regulation of Calcium Dynamics and Propagation Velocity by Tissue Microstructure in Engineered Strands of Cardiac Tissue. Integr. Biol. 12 (2), 34–46. 10.1093/intbio/zyaa003 PMC11956900 32118279

[B48] Rao L. Qian Y. Khodabukus A. Ribar T. Bursac N. (2018). Engineering Human Pluripotent Stem Cells into a Functional Skeletal Muscle Tissue. Nat. Commun. 9 (1), 126. 10.1038/s41467-017-02636-4 29317646 PMC5760720

[B49] Rybakova I. N. Amann K. J. Ervasti J. M. (1996). A New Model for the Interaction of Dystrophin with F-Actin. J. Cel Biol 135 (3), 661–672. 10.1083/jcb.135.3.661 PMC2121071 8909541

[B50] Rybakova I. N. Ervasti J. M. (1997). Dystrophin-glycoprotein Complex Is Monomeric and Stabilizes Actin Filaments *In Vitro* through a Lateral Association. J. Biol. Chem. 272 (45), 28771–28778. 10.1074/jbc.272.45.28771 9353348

[B51] Santosa K. B. Keane A. M. Jablonka-Shariff A. Vannucci B. Snyder-Warwick A. K. (2018). Clinical Relevance of Terminal Schwann Cells: An Overlooked Component of the Neuromuscular junction. J. Neuro Res. 96 (7), 1125–1135. 10.1002/jnr.24231 PMC6292684 29536564

[B52] Santoso J. W. McCain M. L. (2021). Engineering Skeletal Muscle Tissues with Advanced Maturity Improves Synapse Formation with Human Induced Pluripotent Stem Cell-Derived Motor Neurons. NCBI Gene Expr. Omnibus (Geo). 10.1063/5.0054984 PMC8282350 34286174

[B53] Santoso J. W. Li X. Gupta D. Suh G. C. Hendricks E. Lin S. (2021). Engineering Skeletal Muscle Tissues with Advanced Maturity Improves Synapse Formation with Human Induced Pluripotent Stem Cell-Derived Motor Neurons. APL Bioeng. 5 (3), 036101. 10.1063/5.0054984 34286174 PMC8282350

[B54] Santoso J. W. Li X. Gupta D. Suh G. C. Hendricks E. Lin S. (2021). Engineering Skeletal Muscle Tissues with Advanced Maturity Improves Synapse Formation with Human Induced Pluripotent Stem Cell-Derived Motor Neurons. APL Bioeng. 5 (3), 036101. 10.1063/5.0054984 34286174 PMC8282350

[B55] Santoso J. W. McCain M. L. (2020). Neuromuscular Disease Modeling on a Chip. Dis. Models Mech. 13 (7), dmm044867. 10.1242/dmm.044867 PMC7358135 32817118

[B56] Sheikh O. Yokota T. (2021). Developing DMD Therapeutics: a Review of the Effectiveness of Small Molecules, Stop-Codon Readthrough, Dystrophin Gene Replacement, and Exon-Skipping Therapies. Expert Opin. Investig. Drugs 30 (2), 167–176. 10.1080/13543784.2021.1868434 33393390

[B57] Speciale A. A. Ellerington R. Goedert T. Rinaldi C. (2020). Modelling Neuromuscular Diseases in the Age of Precision Medicine. Jpm 10 (4), 178. 10.3390/jpm10040178 PMC7712305 33080928

[B58] Suh G. C. Bettadapur A. Santoso J. W. McCain M. L. (2017). Fabrication of Micromolded Gelatin Hydrogels for Long-Term Culture of Aligned Skeletal Myotubes. Methods Mol. Biol. 1668, 147–163. 10.1007/978-1-4939-7283-8_11 28842908

[B59] Uzel S. G. M. Platt R. J. Subramanian V. Pearl T. M. Rowlands C. J. Chan V. (2016). Microfluidic Device for the Formation of Optically Excitable, Three-Dimensional, Compartmentalized Motor Units. Sci. Adv. 2 (8), e1501429. 10.1126/sciadv.1501429 27493991 PMC4972469

[B60] van der Wal E. Herrero-Hernandez P. Wan R. Broeders M. in 't Groen S. L. M. van Gestel T. J. M. (2018). Large-Scale Expansion of Human iPSC-Derived Skeletal Muscle Cells for Disease Modeling and Cell-Based Therapeutic Strategies. Stem Cel. Rep. 10 (6), 1975–1990. 10.1016/j.stemcr.2018.04.002 PMC5993675 29731431

[B61] Verhaart I. E. C. Johnson A. Thakrar S. Vroom E. De Angelis F. Muntoni F. (2019). Muscle Biopsies in Clinical Trials for Duchenne Muscular Dystrophy - Patients' and Caregivers' Perspective. Neuromuscul. Disord. 29 (8), 576–584. 10.1016/j.nmd.2019.06.004 31378431

[B62] Wang R. T. Barthelemy F. Martin A. S. Douine E. D. Eskin A. Lucas A. (2018). DMD Genotype Correlations from the Duchenne Registry: Endogenous Exon Skipping Is a Factor in Prolonged Ambulation for Individuals with a Defined Mutation Subtype. Hum. Mutat. 39, 1193–1202. 10.1002/humu.23561 29907980 PMC6175390

[B63] Wein N. Vulin A. Falzarano M. S. Szigyarto C. A.-K. Maiti B. Findlay A. (2014). Translation from a DMD Exon 5 IRES Results in a Functional Dystrophin Isoform that Attenuates Dystrophinopathy in Humans and Mice. Nat. Med. 20 (9), 992–1000. 10.1038/nm.3628 25108525 PMC4165597

[B64] Wein N. Vulin A. Findlay A. R. Gumienny F. Huang N. Wilton S. D. (2017). Efficient Skipping of Single Exon Duplications in DMD Patient-Derived Cell Lines Using an Antisense Oligonucleotide Approach. Jnd 4 (3), 199–207. 10.3233/JND-170233 28869484

[B65] White J. Barro M. V. Makarenkova H. P. Sanger J. W. Sanger J. M. (2014). Localization of Sarcomeric Proteins during Myofibril Assembly in Cultured Mouse Primary Skeletal Myotubes. Anat. Rec. 297 (9), 1571–1584. 10.1002/ar.22981 PMC4145531 25125171

[B66] Wilton S. D. Fall A. M. Harding P. L. McClorey G. Coleman C. Fletcher S. (2007). Antisense Oligonucleotide-Induced Exon Skipping across the Human Dystrophin Gene Transcript. Mol. Ther. 15 (7), 1288–1296. 10.1038/sj.mt.6300095 17285139

