# Supplementary figures and images for: Modeling Patient-Specific Muscular Dystrophy Phenotypes and Therapeutic Responses in Reprogrammed Myotubes Engineered on Micromolded Gelatin Hydrogels
Source: Front Cell Dev Biol. 2022 Apr 6;10:830415. doi: 10.3389/fcell.2022.830415 (PMC9020228; doi:10.3389/fcell.2022.830415)

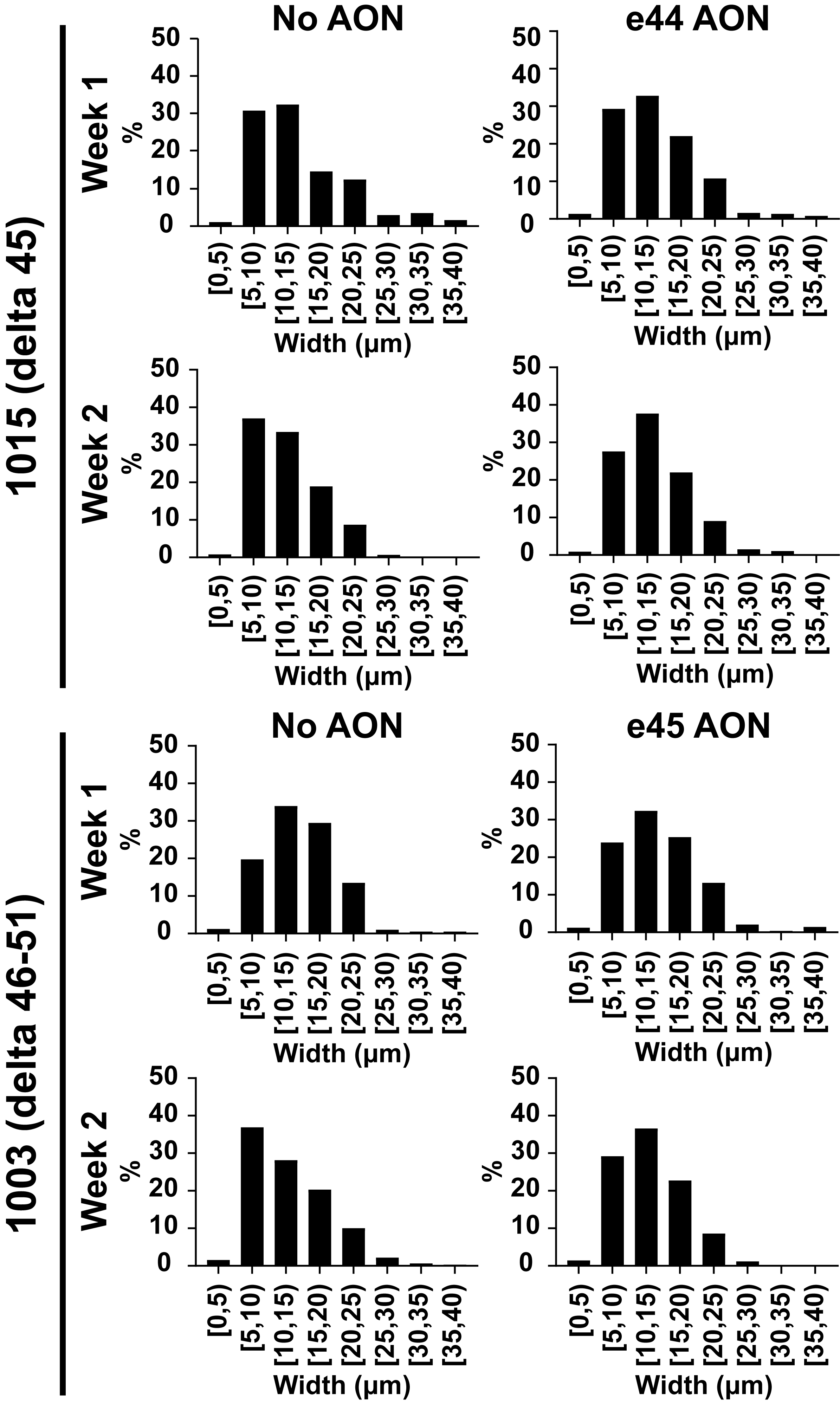

Supplement: Supplementary file 1 [file Image2.TIF]

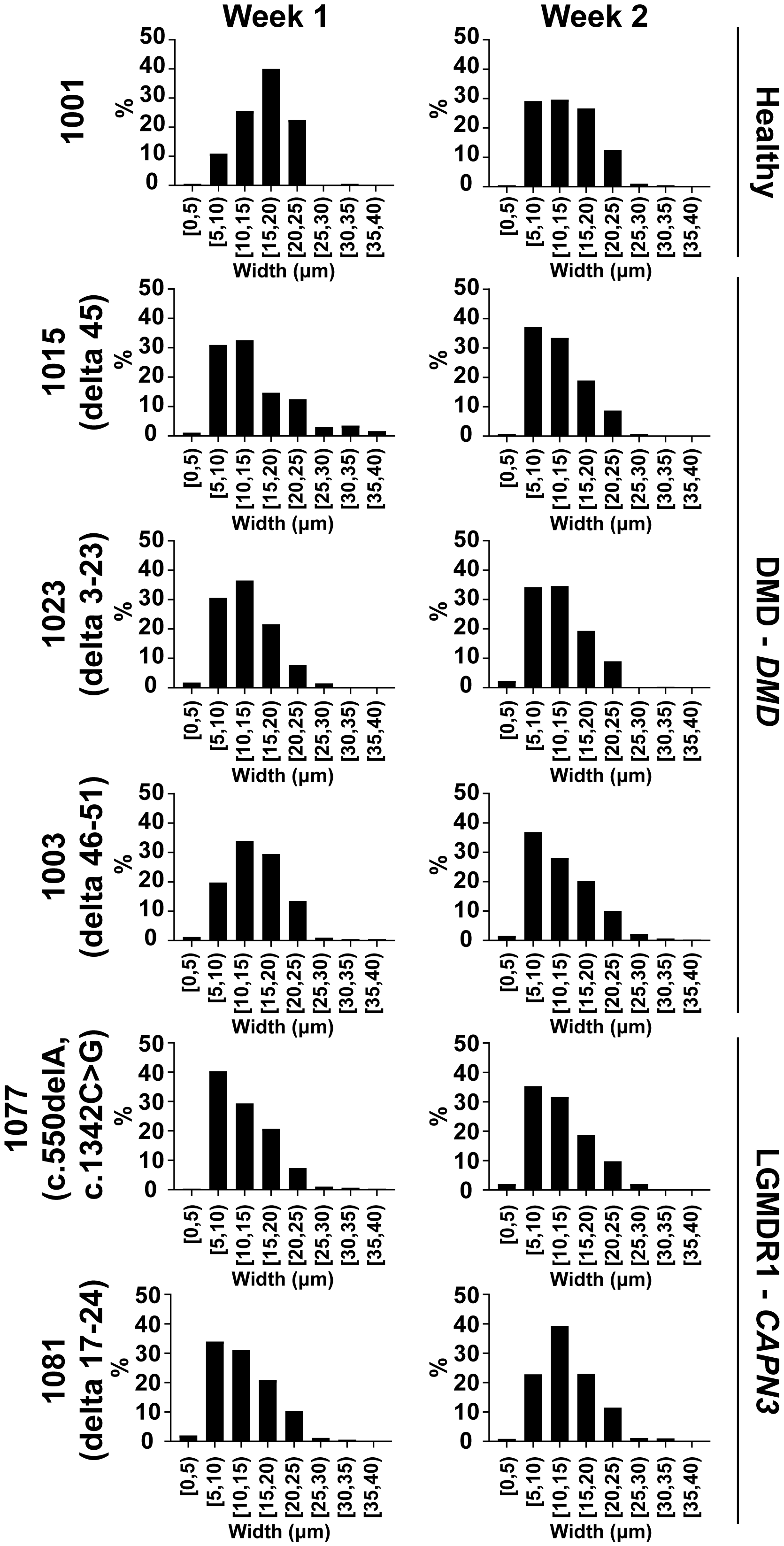

Supplement: Supplementary file 2 [file Image1.TIF]
